# Spontaneous Pregnancy After Surgical Repair of Subseptate Uterus: A Case Report and Review of the Available Literature

**DOI:** 10.7759/cureus.43399

**Published:** 2023-08-13

**Authors:** Ioanna Lamari, Apostolos C Ziogas, Ioannis Thanasas, Konstantinos V Kotronis, Emmanouil M Xydias

**Affiliations:** 1 Department of Internal Medicine, Gennimatas General Hospital, Athens, GRC; 2 Faculty of Medicine, University of Thessaly, School of Health Sciences, Larissa, GRC; 3 Department of Obstetrics and Gynaecology, IASO Thessalias Hospital, Larissa, GRC; 4 Department of Obstetrics and Gynecology, General Hospital of Trikala, Trikala, GRC; 5 Department of Obstetrics and Gynaecology, EmbryoClinic IVF, Thessaloniki, GRC

**Keywords:** congenital uterine abnormalities, uterine factor infertility, metroplasty, operative hysteroscopy, septate uterus

## Abstract

Septate uterus is the most common congenital uterine malformation. It has been associated with poor reproductive outcomes, such as infertility and recurrent miscarriage, in the context of both assisted and non-assisted reproduction, though the exact underlying pathophysiological reasons remain unclear. Diagnosis is based on two-dimensional and three-dimensional ultrasound, magnetic resonance imaging, or laparoscopic/hysteroscopic findings. Hysteroscopic repair of the uterine septum has been shown to confer several benefits to reproductive outcomes, though this fact remains in question, due to inconsistent and or low-quality evidence in the medical literature. An individualized approach to the treatment of infertility patients with septate uteri is imperative, given the plethora of possible underlying factors that may complicate management. In this report, we present the case of a patient with a subseptate uterus and a history of infertility, who, following hysteroscopic metroplasty, managed to conceive and ultimately successfully deliver a healthy child.

## Introduction

Congenital uterine abnormalities are a relatively common condition, affecting 4.3-5.5% of women worldwide [1,2] and encompass a wide range of different anatomical anomalies of the uterus and endometrial cavity. Septate uterus is the most common variant, accounting for approximately 35% of all diagnosed anomalies [1,3]. It is believed to be caused by defective canalization of the uterine cavity via the incomplete reabsorption of the midline uterine septum during embryonic development [1,2]. Uterine septa have been known to demonstrate variable morphology, with partial or incomplete septa being very common [1,3]. The presence of a septate uterus has been associated with infertility and poorer reproductive outcomes [1-3] and has been shown to be more prevalent than other uterine abnormalities in women with infertility [2], although the definitive underlying pathophysiological cause for this association still remains unclear [4].

Initial suspicion or diagnosis of septate or subseptate uterus is typically done using hysterosalpingography or two-dimensional (2D) ultrasonography, with three-dimensional (3D) ultrasonography and MRI being the more advanced methods of diagnosis and classification [5]. However, combined laparoscopy and hysteroscopy remain the most suitable method to both verify the diagnosis and treat the abnormalities via metroplasty [1,5]. Metroplasty via hysteroscopic resection with or without laparoscopic guidance is the standard treatment approach, with the primary aim of improving fertility and obstetrical outcomes, while ensuring safe surgical outcomes as well, via direct laparoscopic guidance. However, the diagnostic and therapeutic effectiveness of this intervention has been questioned, with contradictory data and differing expert opinions. The 2016 American Society for Reproductive Medicine (ASRM) guidelines recommend septum removal [6], the 2018 guideline on recurrent pregnancy loss by the European Society of Human Reproduction and Embryology (ESHRE) does not [7], and the Royal College of Obstetricians and Gynaecologists (RCOG) recommends it, with the stipulation that it should be performed by experienced specialists [8].

In light of such differences in practice recommendations, the importance of an individualized approach based on patient circumstances and characteristics is highlighted. In this report, we present the case of a patient with subseptate uterus, infertility, and early pregnancy loss, who underwent hysteroscopic metroplasty under laparoscopic guidance and ultimately succeeded in delivering a healthy child. We also discuss the merits and limitations of the approach followed through a review of the current literature.

## Case presentation

A 37-year-old female of normal BMI, gravida 0, para 0, and her 38-year-old partner presented to our fertility clinic with primary infertility for the past 18 months, despite active attempts at conception. Her menstrual cycles were regular and she did not report dysmenorrhea, menorrhagia, or sexual dysfunctions. Her medical history most notably included hypothyroidism and panic disorder, for which she had been receiving medical treatment (levothyroxine supplementation and a combination of venlafaxine and alprazolam, respectively), as well as a prior chlamydia infection. Initial laboratory infertility investigation showed that her hormonal profile was within the normal range for her age, except for anti-Mullerian hormone (AMH) levels, which were below the expected values.

The patient’s partner was a 38-year-old healthy man with no reported loss of libido, other intercourse dysfunctions, or urinary tract symptoms that may indicate male factor infertility. While he stated that he smoked and occasionally consumed alcohol in social settings, basic semen analysis revealed normal sperm parameters. Karyotyping and cystic fibrosis testing revealed no abnormal findings.

**Figure 1:**
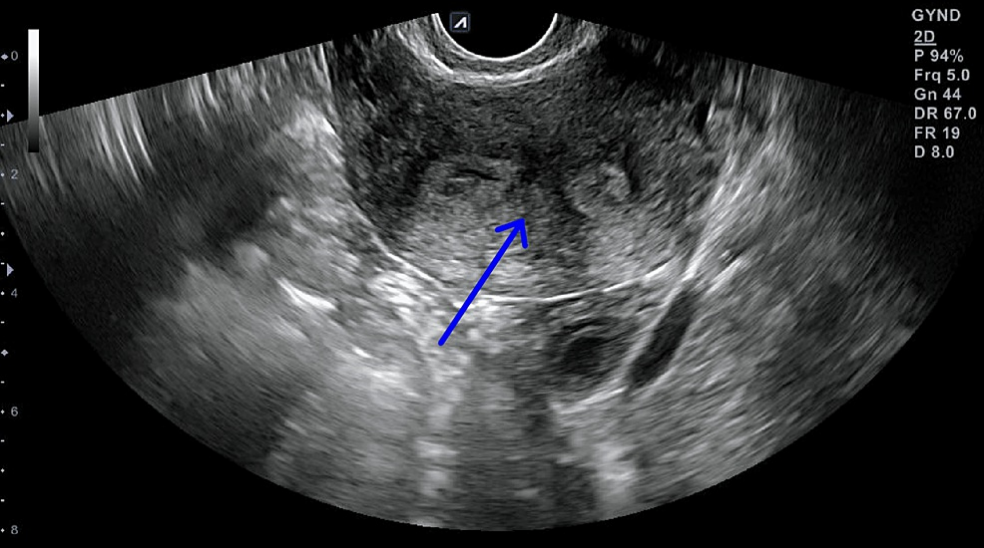
Two-dimensional ultrasound indicates uterine septum.

**Figure 2:**
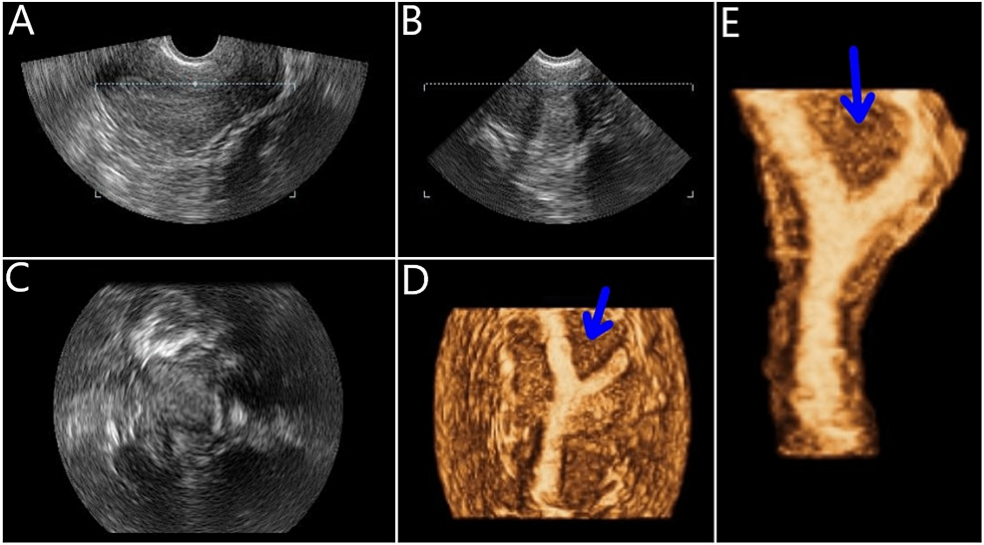
Three-dimensional transvaginal ultrasound scans confirm the clinical suspicion of subseptate uterus. (A) Midsagital plane; (B) Transversal plane; (C) Coronal plane; (D) 3D reconstruction of the uterus; (E) Focused 3D reconstruction of the endometrium

**Figure 3:**
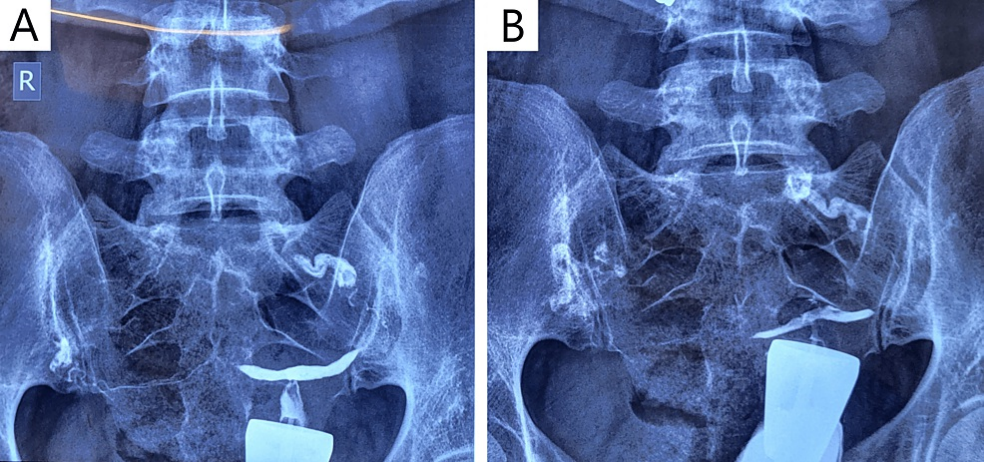
Sequential x-ray hysterosalpingography images indicating normal fallopian tube patency.

Infertility investigation continued with imaging modalities. On 2D transvaginal ultrasound (TVUS) examination, the patient was shown to have good ovarian reserves (antral follicle count = 16); however, suspicion of a congenital uterine abnormality was raised (Figure 1), with a septate or bicornuate uterus being the most likely diagnosis. A supplementary 3D TVUS was performed to further investigate the uterine cavity, ultimately revealing a uterine septum (Figure 2). The patient additionally underwent hysterosalpingography to investigate fallopian tube patency and/or hydrosalpinx, with findings ultimately being normal (Figure 3). Finally, genetic infertility investigation was performed and karyotyping, cystic fibrosis genetic testing, and thrombophilia testing were ordered. Findings were normal with the exception of MTHFR double heterozygosity for C677T and A1298C mutations.

**Figure 4:**
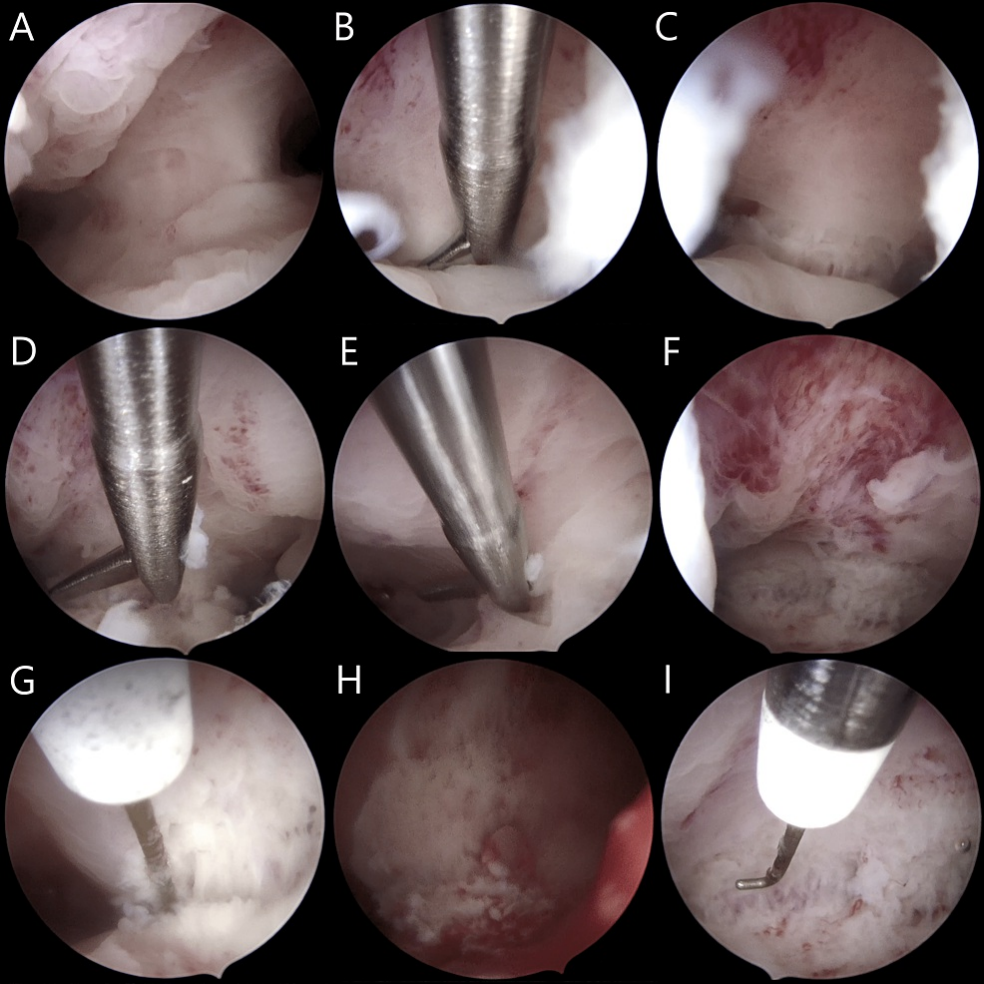
Hysteroscopic metroplasty (septal resection). (A) The septum is located and adequately visualized with the hysteroscopic camera; (B-F) Initial approach with the hysteroscopic scissors. Initialization of a transverse incision on the septum, parallel to the uterine fundus; (G-H) Septal resection continues with the monopolar hook diathermy across the prior transverse incision; (I) Finalization of the septal resection, normal fundal anatomy has been restored.

**Figure 5:**
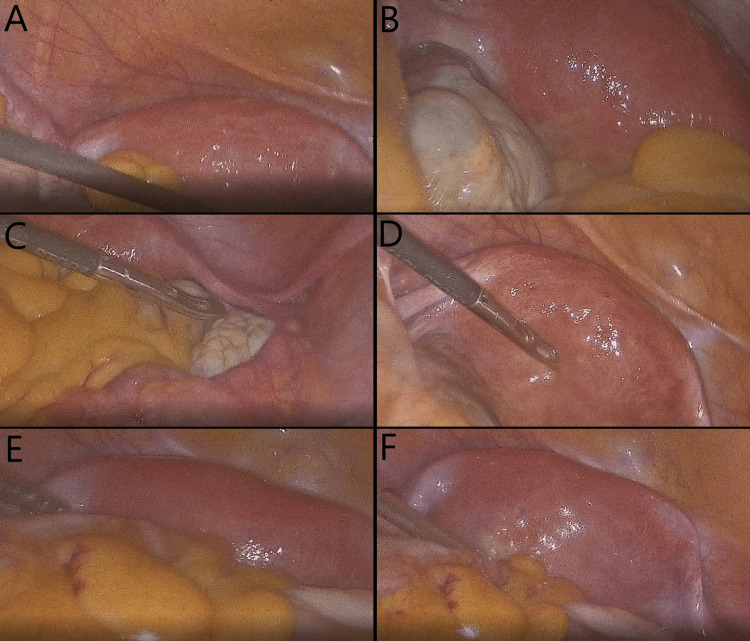
Laparoscopic guidance of the hysteroscopic septal resection. Laparoscopic inspection of the peritoneal cavity for adhesions and other abnormalities and subsequent intra-operative monitoring of the uterine fundus for any signs of injuries or uterine perforation. Images are presented in sequential order (A-F).

A couple of days following the initial ultrasound examination, the patient had a biochemical pregnancy, which, however, did not advance further. Considering the patient's history and data from ultrasonographic investigations, following medical consultation, the patient agreed to undergo hysteroscopic septal resection under laparoscopic guidance before any fertility treatments commenced. During surgery, the uterine septum was identified and the condition was classified as a subseptate uterus. Hysteroscopic metroplasty under laparoscopic guidance was successfully performed without complications (Figures 4, 5).

Three months post-operatively, elevated beta-human chorionic gonadotropin (β-hCG) levels were detected; however, during follow-up examinations, the levels underwent wide fluctuations for more than two weeks, with no ultrasonographic evidence of intrauterine gestational sac. She was subsequently diagnosed with pregnancy of unknown location and, following thorough medical consultation, she consented to methotrexate administration due to the increased risk of ectopic pregnancy.

The patient ultimately received approval for assisted reproductive technology (ART) treatment from the Greek National Authority of Assisted Reproduction and initiated preparations to undergo In vitro fertilization (IVF) treatment. A few months later, the patient reported a spontaneous pregnancy, which proceeded uneventfully and led to a cesarean delivery of a full-term healthy boy.

## Discussion

In this report, we presented the case of a 37-year-old woman with infertility that was most likely largely caused by a uterine septum, since no other major causes of infertility were identified in her or her partner. Following hysteroscopic metroplasty under laparoscopic guidance, the defect was corrected and she managed to conceive. Unfortunately, however, that gestation was diagnosed as pregnancy of unknown location and, following a thorough explanation of the associated risks of continuing, the patient consented to medical termination. Ultimately, following preparation for ART treatment, the patient successfully conceived, carried, and delivered a full-term, healthy boy.

The present case report highlights the challenges and complications that the presence of uterine abnormalities in general, and septate/subseptate uterus (henceforth: septate uterus) in particular, introduces to the investigation and management of infertility patients. Congenital uterine abnormalities have long been associated with poor reproductive outcomes in the medical literature [9]. Although there have been studies reporting normal fertility and reproductive outcomes in such patients as well [10], the general trends in the literature indicate that such abnormalities, particularly septate uterus, are strongly associated with infertility and other adverse reproductive outcomes [1,11], with septate uterus being the most commonly encountered abnormality in females with infertility [2]. Despite the wide acceptance of this association, the exact underlying pathophysiology remains elusive. A recent systematic review concluded that, despite the structural and functional similarity of the septum with normal uterine tissue, there are considerable differences in gene expression, hormonal production, vascularization, and histopathologic features [12]. These differences between normal endometrium and septal endometrium affect hormonal responsiveness and endometrial receptivity and may lead to manifestations of infertility, recurrent pregnancy loss, premature delivery, or other adverse outcomes, which also depend on the individual characteristics of each septum [12].

Given the aforementioned association of septate uterus and infertility, several studies to date have investigated the effect of hysteroscopic repair on reproductive outcomes. In their meta-analysis, Valle et al. demonstrated a pooled pregnancy rate of 67.8% (95%CI, 62.5-72.8), and a live birth rate of 53.5% (95%CI, 47.8-59.1) after hysteroscopic metroplasty for infertility and/or recurrent miscarriage, indicating an improvement in reproductive outcomes, lacking however a comparator group [13]. In a more recent meta-analysis by Carrera et al., the investigators compared the reproductive outcomes of patients that underwent hysteroscopic resection of the uterine septum to expectant management [14]. Overall, the authors demonstrated a statistically significant decrease in miscarriage rate in the intervention groups (OR=0.45, 95%CI, 0.22-0.90), decrease in fetal malpresentation rate (OR= 0.32, 95%CI 0.16 0.65), and, for subseptate uterus in particular, a decrease in preterm birth (OR= 0.30, 95%CI 0.11-0.79) [14]. However, pregnancy and live birth rates had no statistically significant differences between the two groups.

Another meta-analysis by Noventa et al. produced similar results with regard to recurrent miscarriage (OR 0.02, 95%CI 0.02-0.04), preterm labor (OR 0.05, 95%CI 0.03-0.08), with pregnancy rate still demonstrating no statistically significant difference [15]. An additional finding of this study was the statistically significant improvement in live birth rate (OR 3.07, 95%CI 1.22-7.73) in the hysteroscopic metroplasty group. The most recent meta-analysis on the topic by Jiang et al. largely corroborated the findings of the aforementioned investigations but additionally demonstrated an improvement in clinical pregnancy rate (OR 2.28; 95%CI, 1.04-4.98) in the particular patient subgroup that had primary infertility as their indication for surgery [16]. This statistical significance was not maintained when a similar subgroup analysis was conducted for patients with recurrent miscarriage as their primary surgical indication. Rikken et al. conducted the only available randomized control trial (RCT) on the subject to date and demonstrated that there were no statistically significant differences in any reproductive outcomes between patients who underwent hysteroscopic metroplasty and those who received no treatment [3]. However, the authors do concede that the small sample size would only permit the detection of large differences in outcomes, thus smaller, but still significant effects may have been missed. In the current case, metroplasty did manage to improve the odds of pregnancy contrary to the conclusions from the majority of the available literature, but in agreement with the meta-analysis by Jiang et al. [16]. The patient also managed to achieve pregnancy without spontaneous miscarriage, unlike what happened with her biochemical pregnancy prior to surgery, and had a full-term delivery, observations that were consistent with the conclusions from the vast majority of the available literature.

In the present case, the first post-operative attempt at conception resulted in a pregnancy of unknown location, a term that describes a biochemical pregnancy without evidence of an intrauterine gestational sac during TVUS [17]. It may have evolved into an ectopic pregnancy, a condition that endangers the integrity of the patient’s reproductive system, most commonly the fallopian tubes, and is considered life-threatening, mandating emergency treatment [17]. In our case, sequential β-hCG measurements indicated pregnancy and fluctuated throughout the monitoring period without any conclusive ultrasonographic evidence. In such cases, evidence from the medical literature has indicated that active management with methotrexate is significantly more effective in pregnancy resolution and significantly less likely to lead to unscheduled surgery compared to expectant management [18]; therefore, following consultation with the patient, it was ultimately the preferred, safer option.

The patient’s second attempt at conception, following preparation for ART treatment was ultimately successful. While in the present case, the patient conceived spontaneously, evidence from the literature has demonstrated that hysteroscopic metroplasty for septate uterus significantly improves reproductive outcomes of IVF and/or intracytoplasmic sperm injection (ICSI). Ban-Frangez et al. [19] and Tomazevic et al. [20] retrospectively demonstrated that the miscarriage rate of females with septate uterus was significantly decreased, to the level of that of women with a normal uterus, after hysteroscopic metroplasty and IVF/ICSI. Tomazevic et al. additionally demonstrated a significant improvement in pregnancy and live birth rate after metroplasty [20]. From an initial up to seven times lower pregnancy rate and an up to 32 times lower live birth rate, women with septate uterus managed to achieve similar outcomes after hysteroscopic septum repair compared to normal controls, showing no statistically significant differences in pregnancy and live birth rates [20].

Hysteroscopic septal resection is a minimally invasive procedure that has been shown to improve reproductive outcomes, while simultaneously being a simple and safe surgical option, with reduced patient morbidity [21]. However, the lack of evidence from RCTs and the potential for surgical and later maternal complications are factors that should be considered [21,22]. In clinical practice, individual patient circumstances are a considerable factor and always warrant attention and consideration during patient management. In the present case, the patient suffered from primary infertility, with no other reversible factors, apart from her septate uterus. Therefore, minimally invasive surgical repair of that septum was a promising solution, which ultimately resulted in the successful conception and delivery of a healthy child. 

## Conclusions

Septate uterus is a common congenital uterine abnormality, most prevalent in women with recurrent miscarriage and infertility, with the precise underlying mechanism remaining elusive. Hysteroscopic metroplasty with or without laparoscopic guidance is the treatment of choice and is widely applied in clinical practice to improve reproductive outcomes, although data from the available medical literature are yet inconclusive on its efficacy, with contradictory studies and lack of high-quality evidence for either side of the argument. In this report, we demonstrated the efficacy of this intervention in the management of a patient with primary infertility that had no other reversible causes as per her history. Despite a few medical challenges since the procedure, which were ultimately resolved via patient-centered consultation, the patient ultimately succeeded and delivered a healthy child. Further research is required, in particular large RCTs with standardized diagnosis and classification criteria, in addition to standardized surgical methodology, in order to limit confounding factors and reach high-quality results.
